# Methane emissions from tree stems in neotropical peatlands

**DOI:** 10.1111/nph.16178

**Published:** 2019-10-25

**Authors:** Sofie Sjögersten, Andy Siegenthaler, Omar R. Lopez, Paul Aplin, Benjamin Turner, Vincent Gauci

**Affiliations:** ^1^ School of Biosciences The University of Nottingham Sutton Bonington Loughborough LE12 5RD UK; ^2^ Department of Environment, Earth & Ecosystem Sciences STEM Faculty The Open University Milton Keynes MK7 6AA UK; ^3^ Smithsonian Tropical Research Institute Apartado 0843‐03092 Balboa Ancon Republic of Panama; ^4^ Instituto de Investigaciones Científicas y Servicios de Alta Tecnología Edificio 219, Ciudad del Saber, Clayton Panamá Republic of Panama; ^5^ Department of Geography Edge Hill University Ormskirk L39 4QP UK; ^6^ Birmingham Institute of Forest Research (BIFoR) School of Geography, Earth and Environmental Sciences The University of Birmingham Edgbaston Birmingham B15 2TT UK

**Keywords:** greenhouse gases, methane, neotropics, peatlands, tree emissions, tree species, tropical forest

## Abstract

- Neotropical peatlands emit large amounts of methane (CH_4_) from the soil surface, but fluxes from tree stems in these ecosystems are unknown. In this study we investigated CH_4_ emissions from five tree species in two forest types common to neotropical lowland peatlands in Panama.
- Methane from tree stems accounted for up to 30% of net ecosystem CH_4_ emissions. Peak CH_4_ fluxes were greater during the wet season when the water table was high and temperatures were lower. Emissions were greatest from the hardwood tree *Campnosperma panamensis*, but most species acted as emitters, with emissions declining exponentially with height along the stem for all species.
- Overall, species identity, stem diameter, water level, soil temperature and soil CH_4_ fluxes explained 54% of the variance in stem CH_4_ emissions from individual trees. On the landscape level, On the landscape level, the high emissions from *C. panamensis* forests resulted in that they emitted at 340 kg CH_4_ d^−1^ during flooded periods despite their substantially lower areal cover.
- We conclude that emission from tree stems is an important emission pathway for CH_4_ flux from Neotropical peatlands, and that these emissions vary strongly with season and forest type.

## Introduction

Wetlands are the largest natural source of atmospheric methane (CH_4_) (Houghton *et al*., 1994; Ciais *et al*., 2013), contributing 17–40% of global emissions (Laanbroek, 2010; IPCC 2013). Two‐thirds of the global CH_4_ emissions from wetlands are estimated to come from undisturbed tropical wetlands (Melton *et al*., 2013). Of the total area of wetlands in the tropics (1386 000 km^2^), peatlands represent 32%, located mainly in the lowland humid forests of Southeast Asia, Amazonia, Central America and equatorial Africa (Page *et al*., 2011; Sjögersten *et al*., 2014; Dargie *et al*., 2017; Leifeld and Menichetti 2018). Given the large area of tropical peatlands and their capacity for high CH_4_ emissions, at least in the Neotropics (Sjögersten *et al*., 2014; Teh *et al*., 2017; Winton *et al*., 2017; Hoyos‐Santillan *et al*., 2019), tropical peatlands may be important components in the global CH_4_ cycle. Furthermore, these ecosystems are vulnerable to human disturbances with direct implications for atmospheric greenhouse gas concentrations (Couwenberg *et al*.^2010^; Page *et al*., 2011; Lähteenoja *et al*., 2012; Sjögersten *et al*., 2014; Leifeld and Menichetti 2018).

Recent evidence points towards trees as an underestimated contributor to wetland CH_4_ emissions and a major pathway for the egress of soil‐produced CH_4_ (Terazawa *et al*., 2007; Gauci *et al*., 2010; Rice *et al*., 2010; Pangala *et al*., 2013, 2015, 2017; Terazawa *et al*., 2015; Covey & Megonigal, 2019; Jefferies *et al*., 2019). This appears to be particularly important in tropical wetlands, because CH_4_ emissions from seasonally flooded wetlands on mineral soils are responsible for half of all CH_4_ emissions from mineral soils within the Amazon basin (Pangala *et al*., 2017). For a tropical forested peatland in Southeast Asia, in the only study so far to report CH_4_ emissions from trees in tropical peatlands (Pangala *et al*., 2013), tree stems were found to emit substantially more CH_4_ than peat surfaces, accounting for 62–87% of the total ecosystem CH_4_ emissions.

Methane production and subsequent emissions from wetland soils are controlled by a number of abiotic and biotic factors linked to the degree of water logging, temperature, substrate availability and microbial community composition (Jauhiainen *et al*., 2005; Wright *et al*., 2011; Hoyos‐Santillan *et al*., 2016; Sjögersten *et al*., 2018; Girkin *et al*., 2018). Vegetation alters all of these factors, either directly or indirectly. Specifically, the vegetation alters microtopography and peat structure, which affects the water table; shading from the canopy, which controls air temperature; and through litter quality and root exudates, which affects substrate availability (Hoyos‐Santillan *et al*., 2016; Baird *et al*., 2017; Girkin *et al*., 2018). In some areas, methane fluxes are controlled by seasonal variation in rainfall (Teh *et al*., 2017), but such impacts depend on the strength of seasonality. Indeed, work in the Bocas del Toro region, Panama, indicates no clear differences in CH_4_ emissions between seasons, despite water table being a strong predictor of emissions (Wright *et al*., 2011; Hoyos‐Santillan *et al*., 2019).

The magnitude of emissions from trees has been related to tree age, height, density of lenticels, and the concentration of CH_4_ in the soils (Pangala *et al*., 2013, 2015, 2017; Covey & Megonigal, 2019; Welch *et al*., 2019). Tree species differ in their physiology, so the composition of the tree community might also influence tree methane emissions (Pangala *et al*., 2013, 2017). In Neotropical peatlands, peat surfaces emit variable quantities of CH_4_, with persistently high soil emissions in some forest types (e.g. Wright *et al*., 2011; Hoyos‐Santillan *et al*., 2016, 2019; Teh *et al*., 2017; Winton *et al*., 2017). However, the role of the trees as mediators of soil‐produced CH_4_ emissions in such peatland systems is unknown.

Consequently, as high soil CH_4_ emissions have been recorded in Neotropical peatlands, and data on CH_4_ emissions from trees in peatlands in the Neotropics is lacking, we aimed to understand whether trees are large contributors to net ecosystem emissions in neotropical peatlands, and if so to determine the drivers of tree CH_4_ emissions. To address this knowledge gap we formulated the following hypotheses. Tree emissions are an important contributor to net CH_4_ emissions from tropical peatlands, in line with findings from Southeast Asian peatlands and mineral soil wetland systems (Pangala *et al*., 2017). Tree species, and hence forest species composition, affect the magnitude of tree emissions. To test these hypotheses we measured the contribution of trees to ecosystem CH_4_ emissions in two common hardwood forest types of a well‐studied neotropical lowland peatland in Central America (Panama) using soil and stem sampling chambers coupled to an optical methane analyser along four transects. After examining how the specific stem emissions evolved with stem sampling positions above the forest floor of up to 5 m, we explored how these emissions were influenced by species and by extensions the type of forest stand (mixed or mono‐dominant) and the fluctuations of the water table over contrasting periods of wetness. Finally, land cover mapping allowed us to scale the tree emissions to the two target vegetation types across the whole wetland extent. We focused the study on Central America as it hosts an important number of lowland peatlands (Lawson *et al*., 2014), some of which are well described with regard to vegetation communities and soil CH_4_ emissions (Phillips *et al*., 1997; Sjögersten *et al*., 2011; Wright *et al*., 2011; Hoyos‐Santillan *et al*., 2016).

## Materials and Methods

### Study site

**Figure 1:**
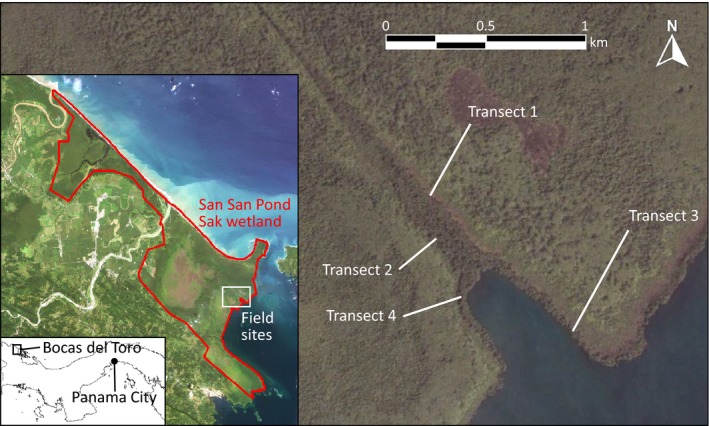
Study site location in Bocas del Toro, Panama, showing extent of the San San Pond Sak wetland overlaid on 2014 RapidEye imagery, and field transects overlaid on 2014 Google Earth imagery.

The study site was in the San San Pond Sak wetland (site 611, rsis.ramsar.org) in the Bocas del Toro Province in the northwestern Caribbean region of Panama (Fig. 1). This wetland covers 164 km^2^ with a range of vegetation types: mangroves, shallow, brackish and freshwater lakes, and peatlands. It includes the 80 km^2^ Changuinola peat deposit, an ombrotrophic peatland within which seven distinct phasic plant communities have been identified (Phillips *et al*., 1997). These communities are palm forest, mixed forest, hardwood forest, stunted forest and open bog plain vegetation covering large areas.

The nearby town of Bocas del Toro, Isla Colon (*c*. 10 km) has a mean annual temperature of 26.4°C (mean min. 26.3°C and mean max 30.3°C) and an annual precipitation average of 3397 mm (Smithsonian Tropical Research Institute physical monitoring programme 2003–2015). The climate is equatorial and there is no distinct dry season. Nevertheless, the meteorological year is divided into four parts: two periods of lower rainfall approximately from mid‐December to the end of May (266 mm per month) and August to October (217 mm per month), and two wetter periods between June and July (329 mm per month) and November to December (403 mm per month) (STRI physical monitoring programme 2004–2015).

We conducted the study in two common phasic communities, mixed forest and *Campnosperma panamensis* forest (Phillips *et al*., 1997), along four independent transects at which both of the forest types occurred (i.e. *n* = 4). The transects were placed on either side of a small inlet into Bahia Almirante to ensure spatial independence (*c*. 900 m apart) (Fig. 1).

We used visual interpretation of very high‐resolution RGB satellite imagery from Google Earth to identify four transects, which included transitions between our two target vegetation types within a relatively short distance (400–600 m). All study sites were freshwater, and further details of the biogeochemical and hydrological properties of the sites can be found elsewhere (Baird *et al*., 2017; Sjögersten *et al*., 2018). At the selected location field data collection was carried out between July 2013 and January 2015.

### Vegetation survey

Along each transect, within each of the target phasic communities we delimited 400 m^2^ plots (this resulted in eight plots in total, i.e. four mixed forest and four *Campnosperma* forest). We subsequently made a vegetation survey of the plots, measuring diameter at breast height for all stems > 3 m in height and identifying them to species based on Phillips *et al*. (1997), the database created by STRI for the Bocas del Toro region ( http://biogeodb.stri.si.edu) and a digital herbarium from the University of Costa Rica ( http://hergol.biologia.ucr.ac.cr).

### Stem‐flux measurements

Stem CH_4_ fluxes were measured from *C. panamensis* (Anacardiaceae), *Cassipourea eliptica* (Rhizophoraceae), *Symphonia globulifera* (Clusiaseae), *Prioria copaifera* (Fabaceae), *Pithecollobium* sp. (Fabaceae) and *Euterpe precatoria* (Palmaceae), which were common species in the study area. The trees selected for measurement were representative of the range of size of mature trees of each species. All species display adaptation to wetland conditions with features such as prop roots, lenticels and aerencyma formation to enable oxygen transport to the root system.

To determine the relationship between stem height and stem CH_4_ emissions and if this varied between species, we measured the stem emissions of the five most abundant hard wood tree species up to 5 m height, *n* = 4 for each species. Measurements were made at 35, 75, 115, 200, 300, 400 and 500 cm. As *E. precatoria* is a small palm it was not possible to measure this species at 400 and 500 cm, and therefore it was not included at this stage.

Tree‐stem CH_4_ emissions were measured using semi‐rigid stem chambers (Siegenthaler *et al*., 2016). The air temperature and air humidity of each chamber was recorded with small data‐loggers (ST‐171, Clas Ohlson, Insjön, Sweden). The chambers were covered with a platicised aluminium foil to maintain adiabatic chamber conditions.

The stem chambers were connected to an ultra‐portable greenhouse gas analyser (UGGA, Los Gatos Research Inc., Mountain View, CA, USA) via two 4.6 m long and 5 mm inside diameter polytetrafluoroethylene‐coated polyvinyl chloride parallel tubes (Nalgene, Rochester, MN, USA) set in a continuous flow mode operating as a closed loop with a flow of 2–4 l min^−1^. The UGGA measured CH_4_ with off‐axis integrated cavity output spectroscopy (OA‐ICOS) at a frequency of 0.33 Hz. Gas concentrations were then measured for 5 min. The analyser's uncertainty in the range of 0.01 to 100 ppmv methane is < 1% without calibration and the precision is ± 0.6 ppb over a period of 100 s (Los Gatos Research, 2013).

The rates were calculated from linear regressions made between the concentration changes starting after an equilibration period of 90 s and the elapsed runtime. After accounting for the chamber volume, which varied between measurements depending on the required chamber size (the range of chamber size was 0.28–1.49 dm^3^ and 95–715 cm^2^ for volume and area, respectively), rates were then expressed relative to the exchange surface area.

The ambient fluxes were corrected to reference fluxes using the following transformation:

(Eqn 1) $$F_{\mathrm{ref}}=F_{\mathrm{amb}}\left[P_{\mathrm{amb}}/P_{\mathrm{ref}}\right]\times \left[T_{\mathrm{ref}}/T_{\mathrm{amb}}\right]$$

where *F*_ref_ = flux corrected to reference conditions, *F*_amb_ = flux measured at ambient conditions, *P*_amb_ = atmospheric pressure at ambient conditions, *P*_ref_ = pressure at reference conditions (1 atm), *T*_ref_ = temperature at reference conditions (298 K) and *T*_amb_ = temperature at ambient conditions (°K).

To find the best‐fit lines going through the seven heights we regressed stem CH_4_ fluxes as a function of height (*F*_CH4_(*h*)) and the stem radius as a function of height (*r*(*h*)). To test if we could predict the fluxes up to 5 m height by only using the first three heights we regressed *F*_CH4_(*h*) and *r*(*h*) with a reduced number of points; for *F*_CH4_(*h*) three heights (35, 75 and 115 cm) and for *r*(*h*) four heights (35, 75, 115 and 200 cm) as the stem circumference were easily measurable up to 200 cm. From the best‐fitted functions established on these first few points we determined which functions best predicted overall emissions up to 5 m high by comparing predicted values with observed emissions.

### Upscaling stem emissions into tree emissions

To determine the tree stem fluxes for the two forest types we measured stem methane fluxes from all trees in each plot between August–October 2013 (a drier period) and November 2014–January 2015 (a wetter period). A total of 297 trees were measured over these two periods, 245 and 52 in the first and second period, respectively. Measurements were balanced between the two vegetation types within each of the two periods. We calculated the stem emissions for a whole tree using *F*_CH4_(*h*) and *r*(*h*). The infinitesimal methane emission (d*F*) from an infinitesimal height (d*h*) could be expressed as:

(Eqn 2) dF=2πf(h)r(h)dh

To calculate the emissions over height range we integrated Eqn 4 over the height going from zero to a certain maximal height *H*. This is done by determining the anti‐derivative (*F*) of the retained *f*(*h*) and *r*(*h*) functions:

(Eqn 3) $$F=2\pi \int_{0}^{H}f\left(h\right)r\left(h\right)dh=F\left(0\right)-F\left(H\right)$$

The two functions that suited all tree species were exponential functions (*f*(*h*) = *ab* ^*h*^ and *r*(*h*) = *cd* ^*h*^). Eqn 4 was reformulated with the anti‐derivative of the two combined functions (Supporting Information Notes S1). This formula was then applied to calculate the stem emissions between ground level (i.e. *h* = 0 m) and the maximum height (i.e. *H* = 5 m). In the few cases where weak negative stem fluxes were recorded, a linear function was used. The anti‐derivative of the two combined functions was:

(Eqn 4) $$F=2\pi \int_{0}^{H}\left(a+bh\right)cd^{h}dh=2\pi c{\left[\left(\frac{b\left(\ln\left(d\right)h-1\right)e^{\ln(d)h}}{\log_{2}\left(d\right)}+\frac{ad^{h}}{\ln(d)}\right)\right]}_{0}^{H}$$

Subsequently we measured tree fluxes for the three lower heights from all trees in a plot at each measurement occasion and integrated stem emissions over 5 m using the established relationships.

### Upscaling from tree emissions to ecosystem stem emissions

After the integration of stem fluxes into tree emissions (*F*_tree_) we averaged the stem emissions per species and plot. To relate stem emissions to the ecosystem surface we multiplied the fluxes by tree density.

### Soil exchange measurements

We determined CH_4_ gas exchange between the soil surface and the atmosphere (*R*_CH4_) adjacent (1 m from the trunk) to *c*. one‐third of measured trees, whcih resulted in 115 soil flux measurements. The day before soil exchange measurement we inserted a lightweight polypropylene rim (inner diameter 30 cm, height 15 cm) 10 cm deep in the soil into the peat surface. For the measurements, we then slotted a cylindrical chamber (diameter 30 cm, height 20 cm) into the rim ensuring an airtight seal. To reduce disturbance of the soil surface, a 120 × 50 cm polystyrene board was used to kneel on during sampling. It is possible that accessing the sampling locations resulted in ebullition. Chamber CH_4_ gas concentration was analysed using the UGGA and fluxes were calculated as described above. All flux series were inspected to ensure ebullition was not affecting the calculated fluxes.

### Water level fluctuations

We monitored water level fluctuations at the plots using a 5 cm diameter perforated PVC dip well inserted into the ground. In addition to the *in situ* water level measurements we used water availability (a standard hydrological parameter based on the water balance) of the soils measured *c*. 10 km away at the research station of the Smithsonian Tropical Research Institute (STRI) in Bocas del Toro as an additional measure to describe the wetness of the soils. Details of this parameter are provided in Notes S2.

### Physicochemical parameters

We collected 200 ml of porewater at a depth of 15 cm with a plastic syringe fitted with a tube that was push into the peat. We then transferred 100 ml to a plastic beaker and measured the dissolved oxygen (DO) with an optical probe, oxido‐reduction potential, electrical conductivity and pH. The probes were connected to a labquest interface (Vernier, Beaverton, OR, USA).

Remaining porewater in the syringe was filtered through 0.2 μm Acrodiscs (Pall, New York, NY, USA). After returning from the field, the tubes were stored at 4°C and shipped in a cooled box within 3 wk for analysis. We filtered 1 ml of the solution through pre‐rinsed 0.45 μm cellulose acetate membrane filters (Whatman, Maidstone, UK) and used an ion chromatograph (Dionex) to measure NO_3_^−^, SO_4_^2−^, Cl^−^, Ca^2+^, Mg^2+^, NH_4_^+^ and K^+^. We measured dissolved organic carbon (DOC) and dissolved nitrogen (TN) using a TOC/TN analyser (Shimadzu, Kyoto, Japan; TOC‐V_CPN_)).

### Land cover maps

To estimate the total volume of CH_4_ emissions for the entire San San Pond Sak wetland area, land cover classification was conducted using high‐spatial‐resolution RapidEye satellite sensor imagery (Notes S3). Methane fluxes from each vegetation type were then scaled to the area of these.

### Statistical analysis

Best‐fit regression models for stem height and CH_4_ fluxes were determined using sigmaplot. We used genstat to run mixed linear models using the residual maximum likelihood (REML) method to investigate the impact of tree species, water levels and stand on CH_4_ fluxes as well as forest stand properties. In the model, species or stand (mixed vs *Campnosperma*) and water level (above or below the peat surface) were used as fixed effects, and transect as the random effect. CH_4_ fluxes were log‐transformed before analysis. Differences in site properties were analysed using REML with stand as the fixed effect and transect as the random effect. Standard error of the differences (SED) were predicted from the mixed linear models.

We investigated the relationship between temperature and gas fluxes using stepwise backward eliminating multiple regression analysis. CH_4_ fluxes were log‐transformed to meet normality assumptions. Normal distributions, homogeneity and homeoscedacity of residuals were checked using QQ‐plots and scatter‐plots for all statistical models to ensure the model residuals met the test assumptions.

## Results

### Forest structure and soil physicochemical properties

The most abundant species in the mixed forest stands were *P. copaifera* and *C. panamensis* followed by *E. precatoria*,* C. eliptica* and *R. taedigera. S. globulifera*,* Pithecollobium* sp. and *Ficus* sp. were also present but at lower density (Table 1). The *Campnosperma* forest was dominated by *C. panamensis* followed by *C. eliptica*, with other species at lower abundance (Table 1). Overall stand height was greatest at the *Campnosperma* forest site while stem density and basal area did not differ significantly among sites (Table 2).

**Table 1:** Forest and tree properties of the species present in mixed and *Campnosperma* forest stands.

Species	Mixed forest				*Campnosperma* forest			
	Density (trees ha^−1^)	Mean basal area (m^2^ per tree)	Species basal area (m^2^ ha^−1^)	Height (m)	Density (trees ha^−1^)	Mean basal area (m^2^ per tree)	Species basal area (m^2^ ha^−1^)	Height (m)
*Campnosperma panamensis*	750 (386)	0.029 (0.0036)	22.9 (5.68)	16.79 (1.24)	1756.25 (684)	0.026 (0.0020)	45.85 (2.00)	17.5 (1.12)
*Symphonia globulifera*	56.25 (16)	0.10 (0.030)	6.96 (4.30)	18.5 (2.51)	81.25 (19)	0.060 (0.021)	4.84 (1.68)	18.07 (1.73)
*Cassipourea eliptica*	383.25 (171)	0.0060 (0.00093)	2.57 (0.88)	–	643.75 (250)	0.00616 (0.00084)	3.97 (1.87)	–
*Euterpe precatoria*	518.75 (122)	0.0066 (0.00060)	3.76 (1.19)	7.89 (1.88)	262.5 (96)	0.0062 (0.00076)	1.63 (0.76)	5.32 (0.79)
*Prioria copaifera*	781.25 (270)	0.0025 (0.00027)	2.58 (0.54)	8.48	250	0.0012 (0.0002)	0.29	–
*Raphia taedigera*	225	0.39 (0.11)	117.74	10.43 (1.49)	150	0.24 (0.15)	35.99	5.24 (2.26)
*Pithecollobium* sp.	168.75 (75)	0.013 (0.0032)	2.85 (0.26)	10.55 (0.47)	100 (50)	0.018 (0.0060)	1.84 (1.06)	–
*Ficus* sp.	25	0.00717	0.24	–	–	–	–	–
Unknown 1	–	–		–	25	0.081	2.03	–

Means (SE) are shown.

**Table 2:** Stand characteristics measured at the mixed and *Campnosperma* forests in 2013 and again in 2014 for a subset of physicochemical parameters.

Parameter	Mixed	*Campnosperma*	SED	*F*	*P*
Year 2013					
Peat depth (m)	2.8	3.6	0.42	3.08	ns
Stand height (m)	10.9	18.7	3.5	4.86	ns
Basal area (m^2^ ha^−1^)	69.1	65.6	23.4	0.03	ns
Tree density (ha^−1^)	2550	3200	1103	0.35	ns
pH	4.2	4.3	0.05	5.83	*0.095*
CND (μS cm^−1^)	21.4	17.9	2.68	1.67	ns
DO (mg l^−1^)	2.6	2.2	0.30	2.26	ns
Ca^2+^ (mg l^−1^)	3.96	2.17	0.91	3.82	ns
Cl^−1^ (mg l^−1^)	245	127	62.8	3.48	ns
Mg (mg l^−1^)	11.7	5.9	3.01	3.78	ns
PO_4_^3−^ (mg l^−1^)	1.99	3.65	1.27	1.71	ns
K^+^ (mg l^−1^)	9.38	4.67	1.09	18.74	**0.023**
S^2−^ (mg l^−1^)	28.6	11.5	3.73	21.11	**0.019**
TN (mg l^−1^)	11.6	11.8	1.82	0.01	ns
DOC (mg l^−1^)	32.2	36.8	1.27	13.24	**0.036**
DOC/TN	4.3	5.4	0.8	1.66	ns
*T*_air_ (°C)	28.2	27.9	0.5	0.36	ns
*T*_soil_ (°C)	26.1	25.9	0.2	1.8	ns
Year 2014					
pH	4.1	4.0	0.006	29.4	**0.012**
CND (μS cm^−1^)	67.7	54.5	2.1	36.95	**0.009**
DO (mg l^−1^)	7.9	7.3	0.1	19.96	**0.021**
Redox (mV)	345	354	4	4.29	ns
*T*_air_ (°C)	28.2	28.4	0.7	0.1	ns
*T*_soil_ (°C)	26.5	26.3	0.3	0.68	ns

Mean and SE of the differences, *F* and *P*‐values relating to differences between ‘stands’ in measured parameters each year are reported. NS, *P* > 0.1; bold, significant difference; italics, *P* = 0.05–0.1. CND, conductivity; DO, dissolved oxygen; TN, total dissolved nitrogen; *T*, temperature.

Peat depth at the sites was *c*. 2.8–3.5 m, pH and conductivity were low, and Cl^−^ and S^2−^ concentrations indicate freshwater conditions (Table 2). There was no difference in macro nutrient content between the two forest types (Table 2). DO varied temporally from between 2–3 mg l^−1^ in the dry season of August 2013 to *c*. 7 mg l^−1^ in the wet season of December 2014. Both DO and conductivity were slightly higher at the mixed than the *Campnosperma* forest sites in December 2014. The high DO concentrations in December 2014 were accompanied by high redox potential (*c*. 350 mV). Photosynthetically active radiation below the canopy was low (<100 μmol m^−2^ s^−1^), reflecting the high canopy closure at both sites, and air and soil temperature were 28 and 26°C, respectively.

### Variation in stem‐level CH_4_ fluxes with height

**Figure 2:**
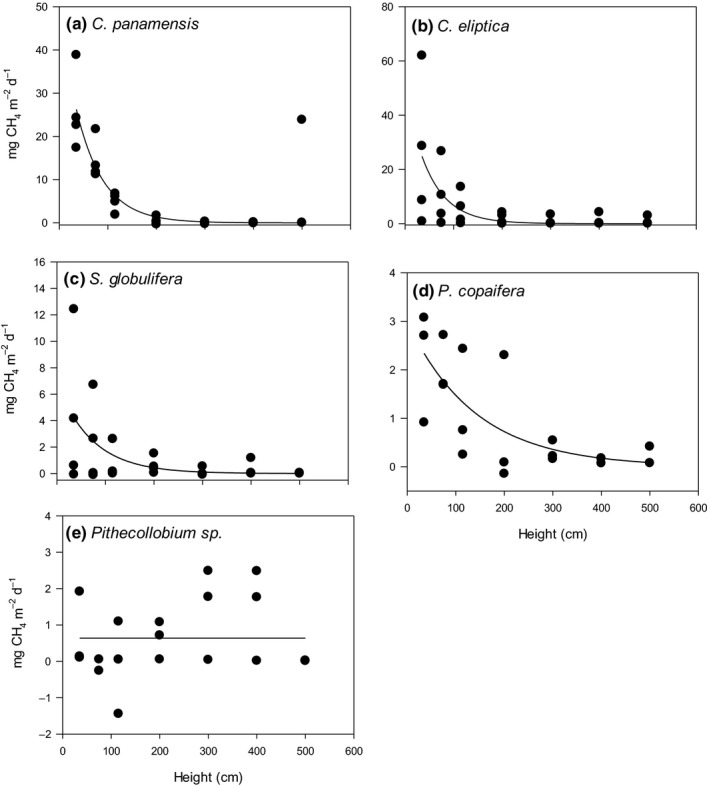
Fitted regression curves between the stem emissions and height of the measurement made for (a) *Campnosperma panamenis*, (b) *Cassipourea eliptica*, (c) *Symphonia globulifera*, (d) *Prioria copaifera* and (e) *Pithecollobium* sp. (*n* = 4). The regression models are reported in Supporting Information Notes S4. [Correction added after online publication 25 October 2019: Fig. 2b and Supporting Information Notes S1 have been updated.]

Methane fluxes were higher closer to the ground, with the greatest fluxes at 35 cm above ground level (Fig. 2). On average, fluxes at 500 cm were 3.5% (0.35 mg m^−2^ d^−1^) of fluxes at 35 cm (9.97 mg m^−2^ d^−1^), while at 115 cm stem emissions were still 26% of those at 35 cm. At 500 cm the proportion of the flux at 35 cm was 0.18% for *C. panamenis*, 3.4% for *C. eliptica*, 5.7% for *S. globulifera*, 8.6% for *P. copaifera* and 54.7% for *Pithecollobium* sp.

### Tree‐level CH_4_ emissions

**Figure 3:**
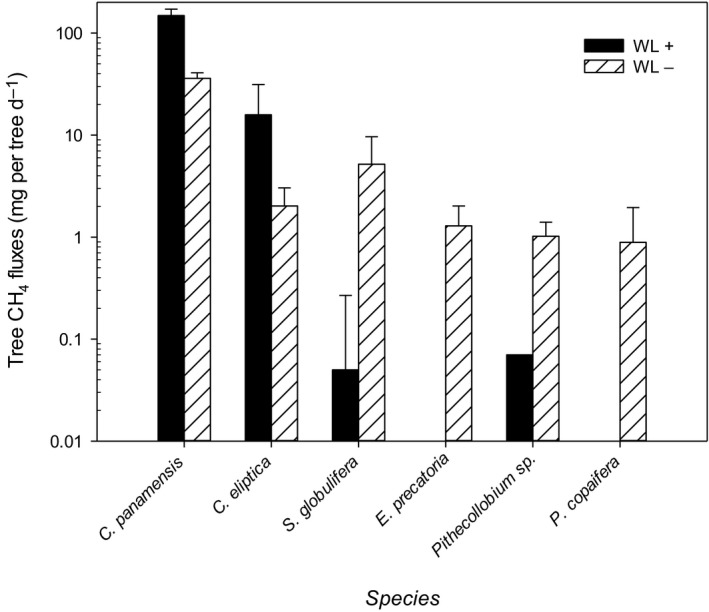
Tree CH_4_ emissions from the six target species integrated to the tree level. Black bars are measurements made when the water table was above the peat surface (WL+) adjacent to the individual tree, while grey bars refer to water tables below the peat surface (WL−). For some locations/trees the water table was always below the peat surface (i.e. there are no black bars for *Euterpe precatoria* or *Prioria copaifera*). Means ± SE are shown (*n* = 32). Note that only one measurement at high water tables was made for *Pithecollobium*.

**Figure 4:**
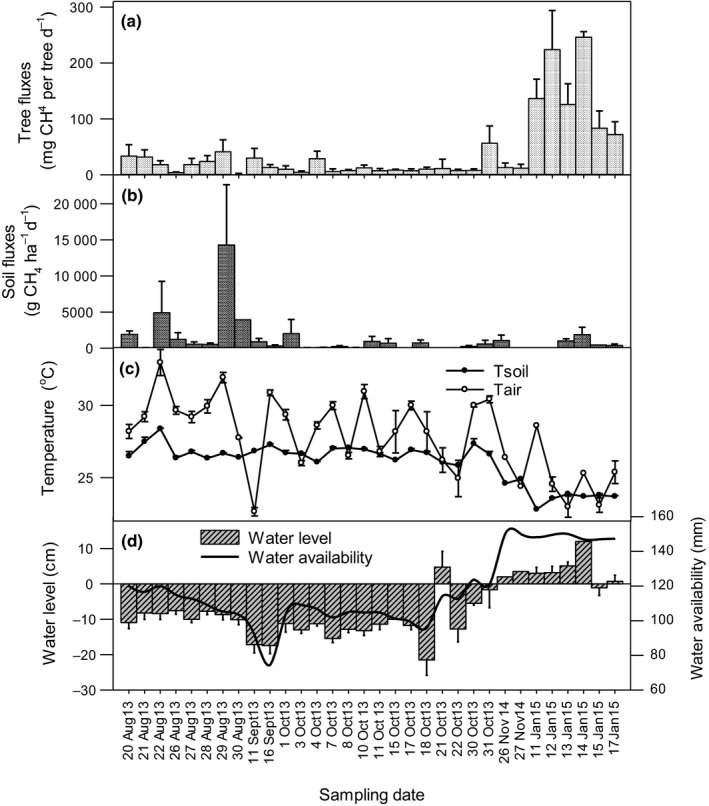
Temporal variation in (a) CH_4_ emissions from trees across the range of tree species and locations measured in a given day, (b) CH_4_ emissions from soil measured adjacent to the trees measured on the same day, (c) soil and air temperature, and (d) water level and water availability. The figure combines all individual measurement dates at the different sites, as the sampling followed a block structure (Fig. 1). Data from each day include both vegetation types and the full range of species. Means ± SE are shown.

Individual tree CH_4_ emissions varied between fluxes near zero to 698 mg per tree d^−1^ and CH_4_ emissions varied strongly among species (*F*_1,291_ = 28.7, *P *<* *0.001). Highest emissions were from *C. panamensis* and *C. eliptica* (Fig. 3) and the lowest emissions were from *Pithecollobium* sp. and *P. copaifera*. Regression analysis showed that 34% of the variance explained was attributed to species. The best model predicting tree CH_4_ fluxes, which explained 54% of the variance, included depth at breast height (DBH), water level, soil temperature and soil CH_4_ fluxes (Fig. 4; Table 3). Emissions were positively related to DBH, higher water levels and soil CH_4_ emissions, and negatively related to soil temperature (wet soils were cooler, Fig. 4).

**Table 3:** Regression models predicting CH_4_ fluxes from individual trees, the soil surface and the ecosystem‐level tree emissions.

Flux type/site	Unit	Parameter	Estimate	df	*F*	*P*	Variance accounted for (%)
Individual trees	Log(CH_4_+10) per tree d^−1^			3, 97	15.35	**<0.001**	30.7
		Constant	3.219				
		Log(CH_4_soil+10)	0.0934				
		*T*_soil_ (°C)	−0.0928				
		DBH (cm)	0.0175				
Soil/mixed forest	Log(CH_4_+10) ha^−1^ d^−1^			3, 17	3.62	**0.04**	31.6
		Constant	14.21				
		*T*_air_ (°C)	0.2049				
		*T*_soil_ (°C)	−0.681				
		WT (cm)^1^	0.0856				
Soil/*Campnosperma*	Log(CH_4_+10) ha^−1^ d^−1^	na		1, 25	0.96	0.3	
Trees/mixed forest	Log(CH_4_+10) ha^−1^ d^−1^			1, 17	11.78	**0.003**	38.8
		Constant	5.27				
		*T*_soil_ (°C)	−0.1502	16			
Tree/*Campnosperma*	Log(CH_4_+10) ha^−1^ d^−1^			1, 25	41.56	**<0.001**	61.9
		Constant	11				
		*T*_soil_ (°C)	−0.3504				

Parameter estimates and overall model statistics are reported. Bold *P*‐values indicate significance. T, temperature; DBH, diameter at breast height; WT, water table; na, not applicable.

^1^Water table (WT) above the peat surface is positive.

Daily averaged tree emissions were greatest in the wetter months of mid‐October 2014 to January 2015, with means of 133 ± 22 mg per tree d^−1^ during times of water levels above the surface, while fluxes were substantially lower with a mean of 20 ± 28 mg per tree d^−1^ during days when the water table was below the peat surface (Fig. 4). Water availability measured at the nearest weather station correlated well with the water level measured in the peatland (*F*_1,300_ = 316.25, *P *<* *0.001, *r* ^2^ = 0.51, water level (cm) = −45.89 + 0.3255 (water availability (mm)) as well as with soil temperature (*r* ^2^ = 0.58, *P *<* *0.0001).

### Ecosystem‐level tree CH_4_ emissions

**Figure 5:**
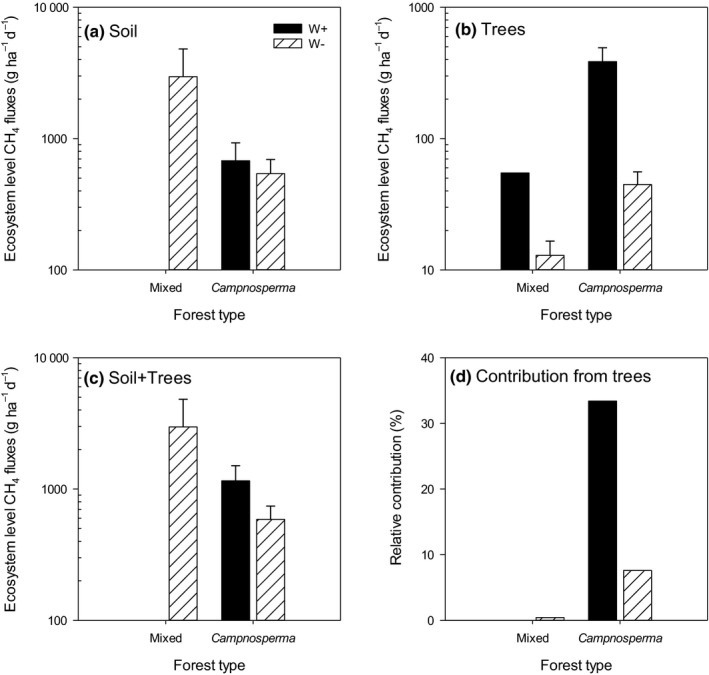
Methane fluxes from (a) soil and (b) trees, (c) net ecosystem fluxes, and (d) relative contribution from trees at mixed and *Campnosperma* forests. Black bars are measurements made when the water table was above the peat surface (WL+) adjacent to the individual tree, while striped bars refer to water tables below the peat surface (WL−). Note the different scale between soil and tree fluxes. Means ± SE are shown.

Soil CH_4_ fluxes were generally large in comparison with stem fluxes, particularly from mixed forest sites (stand: *F*_1,37_ = 4.63, *P *<* *0.05; air and soil temperature were significant covariates in the model: *F*_1,39_ = 7.61; *P *<* *0.01 and *F*_1,39_ = 9.92, *P *<* *0.01, respectively, Fig. 5a). By contrast, tree emissions on the stand level were greatest at *Campnosperma* sites (stand: *F*_1,45_ = 25.64, *P *<* *0.001) with the highest emissions during flooded periods, in line with the fluxes from individual trees (Fig. 5b). The highest net ecosystem emissions (soil+trees) were at the mixed forest site, due to some very high soil CH_4_ emissions, but there was no significant difference between stands (*P *=* *0.1; Fig. 5c). At the *Campnosperma* sites tree emissions accounted for 30% of the net flux during flooded conditions (Fig. 5d).

**Figure 6:**
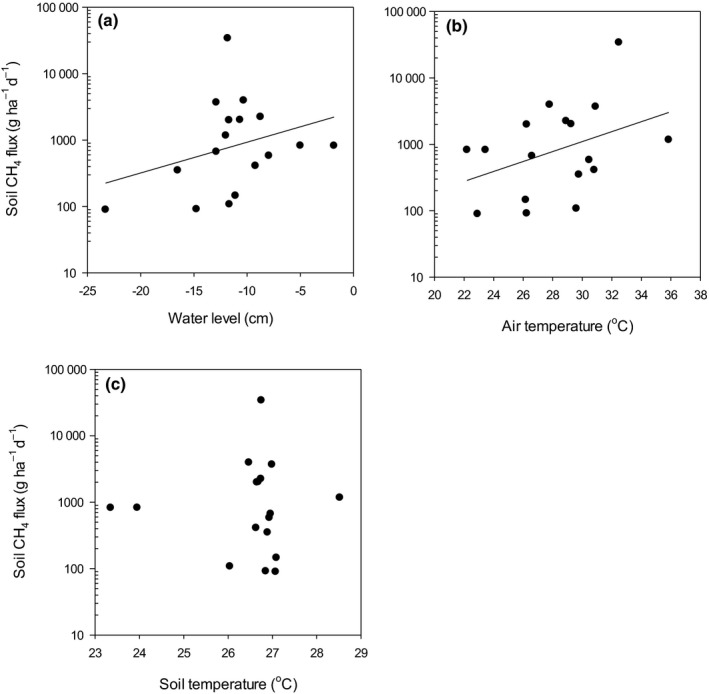
Relationship between the mixed forest site CH_4_ fluxes from the peat surface and (a) water levels (positive values indicate water levels above the peat surface), (b) air temperature and (c) soil temperature. Significant single linear regression lines are plotted; the models are reported in Table 3.

**Figure 7:**
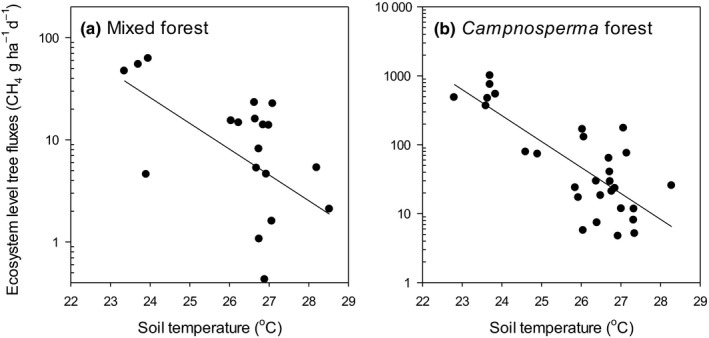
Relationships between estimated ecosystem‐level CH_4_ emissions from trees and soil temperature at (a) the mixed and (b) the *Campnosperma* forest sites. Significant linear regression models are plotted; the models are reported in Table 3.

Soil ecosystem fluxes at the mixed forest site were positively related to water level and air temperature and negatively related to soil temperature, while there was no significant relationship between soil CH_4_ fluxes and any of the environmental variables at the *Camponsosperma* site (Fig. 6; Table 3). Ecosystem‐level tree emissions were negatively related to soil temperature in both of the forest types (Fig. 7; Table 3).

### Upscaling of tree CH_4_ emissions

**Figure 8:**
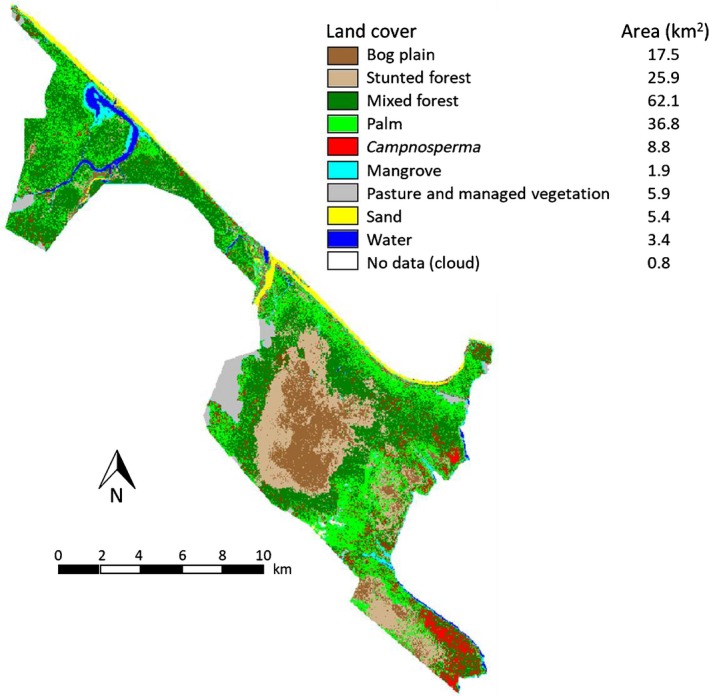
Land cover distribution and areas of different vegetation types across the San San Pond Sak wetland system.

Land cover classification of the wetland vegetation types showed that the two target vegetation stand types, mixed and *C. panamensis*‐dominated forests, covered 62.1 and 8.8 km^2^ of the wetland area, respectively (Fig. 8; Table S1). The other main vegetation types, palm, stunted forest and bog plain, covered 36.8, 25.9 and 17.5 km^2^, respectively. The overall accuracy for the land cover classification was 84.4% (Table S1). When the ecosystem tree fluxes were scaled to the areas of the two forest types, net emissions from mixed and *C. panamensis*‐dominated forests were 340.3 and 339.5 ± 92.7 kg CH_4_ d^−1^, respectively, during the flooded period. Emissions dropped substantially to 80.1 ± 22.8 and 39.3 ± 9.8 kg CH_4_ d^−1^ from mixed and *C. panamensis* forests, respectively, during the period of water tables below the surface.

## Discussion

Our data demonstrate clear differences in both the magnitude and the proportion of tree stem and soil emissions in the two peatland forest communities examined with soil emissions dominating the ecosystem CH_4_ flux. Trees contributed 10–30% of the net ecosystem tree CH_4_ emissions from *C. panamenis* stands, supporting our first hypothesis that tree CH_4_ emissions are an important contributor to net ecosystem CH_4_ fluxes. The high emissions from the *C. panamensis* stands agree with the high contribution of trees to net ecosystem CH_4_ emissions (62**–**87%) in peatlands in Central Kalimantan, Indonesia (Pangala *et al*., 2013), mineral floodplain soils in the Amazon (41–51%) (Pangala *et al*., 2017), and temperate forested wetlands dominated by *Alnus glutinosa* and *Betula pubescens* (27%) (Pangala *et al*., 2015). The contrasting low emissions from the mixed forest site, where tree emissions represented only 0.5% of total ecosystem emissions, suggests strong differences in the contribution made from trees to net emissions between forest types.

Regarding the magnitude of emissions from tropical peatland trees, fluxes from the sites in Panama were much larger (i.e. 386 g ha^−1^ d^−1^) from *C. panamenis* stands (Fig. 5b) than ecosystem‐level tree fluxes reported from the tropical peatlands in Kalimantan, which at 48 g ha^−1^ d^−1^ were in the same range as fluxes from our mixed forest stand and during periods of low water tables at the *C. panamensis* stand (Pangala *et al*., 2013). This suggests that CH_4_ emissions from trees in these neotropical peatlands are significantly lower but of the same order of magnitude as those from mineral soils of the Amazon floodplain, which range between 474 and 2866 g ha^−1^ d^−1^ (Pangala *et al*., 2017) and that tree emissions from neotropical peatlands outside Amazonia also have the potential to be strong CH_4_ sources.

The strong response of ecosystem‐level tree emissions to the water table, soil temperature and stand type suggest that the importance of tree emissions to net ecosystem emissions varies seasonally, and the magnitude of tree contribution to net ecosystem emissions varies across forest types. In previous studies, high soil CH_4_ emissions correlated with high tree emissions (Pangala *et al*., 2013, 2014, 2015). In line with these findings, soil CH_4_ fluxes in our study were positively related to emissions from individual trees (Table 3) and tree emissions were greater during the wet season (Fig. 4). However, this did not hold true on the ecosystem scale. It is likely that the strong negative relationship between soil temperature and ecosystem‐level tree CH_4_ emissions (Fig. 7) is driven by waterlogging (wet soils are cooler, see Fig. 4) and by extension high CH_4_ production. The wider implications of these findings are that forest type and soil temperature could be used to predict tree CH_4_ emissions in tropical peatlands.

Methane emissions from stems varied by several orders of magnitude among species (Fig. 3). This supports results from other temperate and tropical peatland ecosystems, where species‐specific differences were attributed to size, wood and lenticel density (Pangala *et al*., 2013, 2014). Furthermore, pneumatophore density (a species‐level trait) determined peat surface CH_4_ emissions and, by extension, tree emissions in Southeast Asian peatlands (Pangala *et al*., 2013). In our study, stem emissions were greatest from the hardwood tree *C*. *panamesis*; this species forms dense monodominant stands, which explains the high stand‐level CH_4_ emissions from this forest type and suggests that these sites may be hotspots of tree CH_4_ emissions. By contrast, the high emissions from *C. eliptica* and *S. globulifera* stems may be related to their growth habits: *S. globulifera* is a large canopy tree and CH_4_ emissions from individual trees were related to stem diameter, while *C. eliptica* is smaller and in Kalimantan smaller trees emitted more CH_4_ possibly due to greater lenticel densities or lower wood densities in smaller less mature trees (Pangala *et al*., 2013, 2014). Differences in lenticel densities may contribute to differences in tree emissions between the study species. Indeed, *C. panamensis* produced greater lenticel densities near the base of the trunk than *C. eliptica*,* P. copaifera* and *S. globulifera* (O. Lopez, pers. obs.). *S. globulifera* also forms prop roots, an additional adaptation to wetland conditions that might further explain the high CH_4_ stem emissions for this species. At this stage it is not clear if stem CH_4_ emissions from the target species are transported via aerenchyma or through the xylem (Barba *et al*., 2018), and indeed it is possible that the emission pathway contributes to the differences noted among species. In this context it is also important to note that lenticels/prop roots are physiological adaptations to support oxygen transport to the root system under flooded conditions (Kozlowski, 1984; McKee, 1993; Srikanth *et al*., 2016). Improved understanding and quantification of how different plant wetland adaptations impact both oxygen transport into the soil and gas transport back to the atmosphere for different species is critical to enable modelling and upscaling of these processes.

The palm emitted little CH_4_, perhaps because of the structure of its tissue (e.g. the distribution of the vascular bundles within the stems) or its capacity to oxygenate the root zone via extensive aerenchyma formation and reduce soil CH_4_ production and hence the amount of CH_4_ that could potentially be transported through its tissue (Hoyos‐Santillan *et al*., 2016). If the low palm stem emissions are linked to its palm functional traits then this would suggest that stem emissions would not be a large contributor to net ecosystem CH_4_ emissions from palm‐dominated areas, such as the *Mauritia flexuosa*‐dominated peat swamps in the Amazon (Draper *et al*., 2014). Wood density alone does not appear to explain the variation in CH_4_ emission among species, because *C. panamensis* – the highest emitter – has low wood density (0.33 g cm^−3^), yet the other high‐emitters *C. eliptica* and *S. globulifera* have relatively high wood density (0.65 and 0.58 g cm^−3^, respectively).

Plausible explanations for the very high CH_4_ emissions from soil in our study site are high substrate availability in the form of poorly decomposed peat, and root exudates and anaerobic microsites within the upper peat layers resulting in high methanogenic activity, which is not abated by methane oxidation at the surface. High soil CH_4_ emissions from *Camponsperma* stands are driven in part by high DOC concentrations in the soil solution (Wright *et al*., 2013), suggesting substrate limitation of CH_4_ production (Hoyos‐Santillan *et al*., 2016). The importance of substrate quality may also explain the high soil CH_4_ emissions from the mixed forest stand, which has been shown to have higher concentrations of long‐chain *n*‐alkane and high carbohydrate/aromatic ratios in surface peat than *Camposperma* stands (Upton *et al*., 2018). However, incubation studies (Sjögersten *et al*., 2018) suggest comparable rates of CH_4_ production from surface peat in *Camposperma* and mixed forest stands. This indicates that the differences observed in the field are linked to *in situ* variation in abiotic (e.g. water table, redox conditions, temperature, Fig. 6, 7; Table 3) and biotic drivers (e.g. root outputs of exudates and oxygen into the rhizosphere; Hoyos‐Santillan *et al*., 2016; Girkin *et al.,* 2018). The strong temporal differences in soil and tree emissions, with much stronger increases in stem emissions than soil emissions during the wetter period, suggests that trees are especially important transporters of CH_4_ during periods of high water tables. We speculate that this may be due to high CH_4_ production within the root zone during periods of waterlogging that subsequently result in high tree emissions. The lower substrate quality of old peat away from the immediate root zone, together with surface peat oxidation, may explain a less pronounced increase in CH_4_ emissions during periods of high rainfall.

Because trees of different species are able to strongly modify the physicochemistry (pH and redox) and substrate availability (Girkin *et al*., 2019) within their root zone, trees can directly impact soil CH_4_ production and oxidation. Therefore, a dominance of tree species and species physiological adaptation to flooding might modulate soil CH_4_ concentrations and stem emissions and therefore be critical to predict ecosystem‐level CH_4_ emissions in tropical peatlands. If this is the case, understanding the physiological adaptations that impact soil biogeochemistry among different tree species is critical to predict ecosystem‐level CH_4_ emissions.

Despite the much smaller areal extent of the *C. panamensis* stands, trees in this forest type contributed similar amounts of CH_4_ to the atmosphere as the mixed forest during flooded periods and contributed 50% of CH_4_ emissions during drier periods with water tables below the surface. This highlights that a spatially minor vegetation type can be a major contributor to overall tree CH_4_ emissions from an ecosystem. Upscaling of tree stem emissions must therefore consider the full range of vegetation types present in a given area to achieve realistic representation of this emissions pathway.

In conclusion, Neotropical peatlands are strong emitters of CH_4_, emissions from tree stems forming an important component. Stem emissions vary seasonally and among tree species, presenting a source of temporal variability in CH_4_ emissions from these ecosystems. We attribute contrasting tree CH_4_ emissions between the two forest types to: the dominance of the high emitter *C. panamenis*, the high tree density within the *Campnosperma* forest, and degree of waterlogging and magnitude of soil CH_4_ emissions.

## Acknowledgements

We thank Roberto Gonzalez, Bernardo and Martin Lopez, Calvin Cais, Shimara Record, Leonardo Lionel and René Georget for their assistance in the field, staff from the STRI Bocas research station for logistics, Emily Sear for ion chromatography, and Ian Short for mathematical support. This work was supported by Natural Environment Research Council (NERC) grant number NE/J010928/1 to VG and SS and the AXA Research Fund to VG. VG acknowledges support from the NERC consortium MOYA (grant NE/N015606/1).

## Author contributions

SS conceived and designed the study, assisted with site selection, carried out the data analysis and led the writing. AS designed the field study, carried out the fieldwork, calculated the fluxes, carried out initial data exploration, and contributed to the writing. OL contributed to the fieldwork and writing. PA contributed to the fieldwork, carried out the remote sensing component and contributed to the writing. BT contributed to the design of the study, facilitated the logistics of the fieldwork, and contributed to the writing. VG conceived the study, designed the fieldwork, supported the flux calculations and contributed to the writing.

## Supporting information

Please note: Wiley Blackwell are not responsible for the content or functionality of any Supporting Information supplied by the authors. Any queries (other than missing material) should be directed to the *New Phytologist* Central Office.

**Notes S1** Formula used to calculate stem emissions between ground level and maximum height.
**Notes S2** Calculation of water availability.
**Notes S3** Methodology used for land cover classification.
**Notes S4** Fitted regression curves between the stem emissions and height of the measurement.
**Table S1** Error matrix for San San Pond Sak land cover classification.
