# Supplementary material for: Methane emissions from tree stems in neotropical peatlands
Source: New Phytol. 2019 Oct 25;225(2):769–81. doi: 10.1111/nph.16178 (PMC6973267; doi:10.1111/nph.16178)
Supplement: Supplementary file 1 — Notes S1 Formula used to calculate stem emissions between ground level and maximum height. Notes S2 Calculation of water availability. Notes S3 Methodology used for land cover classification. Notes S4 Fitted regression curves between the stem emissions and height of the measurement. Table S1 Error matrix for San San Pond Sak land cover classification. [file NPH-225-769-s001.pdf]

Correction added after online publication on 25 of October: Notes S4 has been updated.

New Phytologist Supporting Information

Title: Methane emissions from tree stems in neotropical peatlands.

Authors: Sofie Sjögersten, Andy Siegenthaler, Omar R. Lopez, Paul Aplin, Benjamin Turner, Vincent Gauci

Article acceptance date: 21 August 2019

Notes 1 Formula used to calculate stem emissions between groundlevel and the maximum height.

$$F = 2p\int_0^H ab^h cd^h \, dh = 2p\left[\frac{ab^h c (b^h)^{\frac{\ln(d)}{\ln(b)}}}{\ln(d)+\ln(b)} + c \right]_0^H$$

Notes 2 Calculation of water availability

This hydro-meteorological data was provided by ETESA (Empresa de Transmisión Eléctrica S.A., Panama, Republic of Panama, www.hidromet.com.pa). As water availability has been monitored daily at STRI since 2010 our aim was to explore if it was a reliable proxy of the water table with the potential to make future predictions of tree CH_4 emissions based on our models. The recalculated maximum field capacity at saturation (water table level = 0) in the peatland was 145 mm. This compares well with the maximum field capacity of 150 mm defined for Panama by the Ministry of Agricultural Development (MIDA, Panama city, Panama).

The hydric balance of the soils was calculated from this formula:

$$\Delta S = P - ET - Q - D \quad (10)$$

Where P is the precipitation, ET is the evapotranspiration measured with the Penman–Monteith equation, Q is the runoff, and D is the drainage through infiltration.

The water availability (WA) is the cumulative value of the water balance (ΔS):

$$\text{Water availability (WA)} = \Sigma\ \Delta S \quad (11)$$

Thereafter, when the water availability (WA) was above the average value (113.5 mm) measured over a period of three years (June 2012 until May 2015) we qualified it as 'high', whereas when WA was below that same average it was qualified as 'low'.

Notes 3 Methodology used for land cover classification

Conventional medium resolution multispectral satellite imagery, such as that provided by the Landsat or Sentinel missions (typically 30 or 20 m resolution), though free of charge, has limited spatial (and in some cases, spectral) detail. This can result in land cover classifications of tropical peatlands lacking thematic detail (only broad classes are used) and/or being relatively inaccurate (Lawson et al., 2014). VHR (very high resolution) imagery can increase accuracy, though the data can be costly and is often affected by cloud cover in tropical regions. Here, a mosaic of two 2012 RapidEye images – the presence of cloud prevented any single image covering the whole study area – was used for analysis. RapidEye imagery is provided at a spatial resolution of 5 m and has five spectral bands covering the blue, green, red, red edge and near infrared parts of the electromagnetic spectrum. The inclusion of a red edge band

is relatively unusual and aids in particular in distinguishing between different types of vegetation. The images were mosaiced using a radiometric balancing process to ensure spectral consistency and the mosaiced data set was cropped to the San San Pond Sak footprint using a boundary shapefile accessed from protectedplanet.net, originally provided by Autoridad Nacional del Ambiente, Panama. A land cover classification system was determined, based largely on the vegetation toposequence identified by Phillips et al. (1997), but also following field reconnaissance and land cover survey conducted throughout the wetland, consultation with local expert stakeholders, preliminary image analysis and with reference to high spatial resolution satellite imagery available through Google Earth. Nine land cover categories were identified for classification, including six classes that broadly correspond to the vegetation toposequence: (1) bog plain, (2) stunted forest, (3) mixed forest, (4) palm forest, (5) *Campnosperma* forest and (6) mangrove (the two separate mangrove classes specified in Phillips et al. (1997) were grouped together here). The remaining three classes were (7) pasture and managed vegetation (predominantly grassland, but some cropped fields), (8) sand and (9) water. Finally, small areas of (10) cloud were masked out as no data.

Reference data sets, collected from field survey and supplemented by observation of VHR imagery available through Google Earth and expert knowledge provided by local stakeholders, were used to both train the classifier and assess the accuracy of the classification. The reference data were divided into separate training and testing data sets to avoid bias.

Initially, many-class (100 classes) unsupervised classification was conducted as part of image familiarisation and as a step towards mapping non-vegetation classes and areas of no data (cloud). One by one observation of these 100 spectral class groupings enabled straightforward identification of sand and water features, and areas of cloud. The sand and water classes were mapped, and together with areas of no data, were masked out from subsequent vegetation classification.

An attempt at supervised classification of all remaining vegetation classes proved relatively inaccurate. One particular problem here was spectral confusion between each of the mangrove and pasture (and other agriculture) classes and the other peatland vegetation classes. Although mangrove and pasture are spectrally similar to other vegetation classes, they are spatially constrained and relatively easy to identify around the periphery of the wetland area – mangroves are present along the coastline and tidal rivers, and regular agricultural parcels cleared from the forest are scattered along the inland boundary. Thus, the footprints of these two classes were digitised manually, mapped, and masked out from subsequent vegetation classification.

Finally, traditional supervised classification was conducted on the remaining five peatland vegetation classes (bog plain, stunted forest, mixed forest, palm forest and *Campnosperma* forest. First, training polygons were identified – between 21 and 41 relatively small polygons, distributed throughout the study area, were identified for each of the classes. The separability of training classes was then evaluated using spectral plots and transformed divergence analysis, and was noted as being broadly acceptable. Some spectral overlap was evident, in particular between stunted, mixed and *Campnosperma* forest, but this is inevitable given the spectral characteristics of these similar vegetation categories. As such, some iteration to training and classification preparation was conducted to achieve the optimal classification outcome. Maximum likelihood classification was conducted and a final, comprehensive land cover map of San San Pond Sak was created by integrating the five peatland vegetation classes with the sand, water, mangrove and pasture classes. The final step was accuracy assessment, whereby an equalized random sample of points (an equal number of points per class) was cross-referenced between the land cover classification and the reference data.

Correction added after online publication on 25 of October: Notes S4 has been updated.

Notes 4 Fitted regression curves between the stem emissions and the height of the measurement.

Best fit regressions models between stem height and stem CH4 fluxes as shown in Figure 2 for the five hard word tree species. The best fit model was an exponential decay model f = a*exp(-b*x)

a) C. panamanesis

$F_{1,26} = 53.34$, $P < 0.0001$, $R^2 = 0.68$

	**Coefficient**	**Std. Error**	**t**	**P**
a	49.15	11.42	4.30	<0.001
b	0.018	0.0045	3.96	<0.001

b) C. eliptica

$F_{1,27} = 17.26$, $P < 0.001$, $R^2 = 0.40$

	**Coefficient**	**Std. Error**	**t**	**P**
a	50.07	22.56	2.22	<0.0001
b	0.020	0.0093	2.17	<0.0001

c) *S. globulifera*

$F_{1,25} = 9.26$, $P < 0.01$, $R^2 = 0.28$

	**Coefficient**	**Std. Error**	**t**	**P**
a	6.84	3.35	2.04	= 0.05
b	0.014	0.0083	1.65	= 0.1

d) *P. copaifera*

$F_{1,19} = 21.91$, $P < 0.01$, $R^2 = 0.54$

	**Coefficient**	**Std. Error**	**t**	**P**
a	2.99	0.66	4.53	<0.001
b	0.0071	0.0025	2.84	=0.01

e) *Pithecollobium* sp.

$F_{1,18} = -1.42*10^{-8}$, $P = 1.0$, $R^2 = -8.34 *10^{-10}$

	**Coefficient**	**Std. Error**	**t**	**P**
a	0.6370	0.4381	1.4541	0.1641
b	$1.91*10^{-11}$	0.0025	$7.64*10^{-9}$	1.0000

Table S1. Error matrix for San San Pond Sak land cover classification.

Predicted class	Reference class									Users accuracy (%)
	Bog plain	Stunted forest	Mixed forest	Palm	Campno-sperma	Man-grove	Pasture/ man. veg	Sand	Water	
Bog plain	20	2	0	1	0	0	1	1	0	80.0
Stunted forest	1	18	4	2	0	0	0	0	0	72.0
Mixed forest	0	1	19	4	1	0	0	0	0	76.0
Palm	0	1	4	19	1	0	0	0	0	76.0
Campno-sperma	0	1	5	3	15	0	1	0	0	60.0
Mangrove	0	0	0	0	0	25	0	0	0	100.0
Pasture/ man. veg	0	0	0	0	0	0	25	0	0	100.0
Sand	0	0	0	0	0	0	1	24	0	96.0
Water	0	0	0	0	0	0	0	0	25	100.0
Producers accuracy (%)	95.2	78.3	59.4	65.5	88.2	100.0	89.3	96.0	100.0	
Overall classification accuracy = 84.4%										

References

Lawson IT, Kelly TJ, Aplin P, Boom A, Dargie G, Draper FCH, Hassan PNZBP, Hoyos-Santillan J, Kaduk J, Large D, et al. 2014. Improving estimates of tropical peatland area, carbon storage, and greenhouse gas fluxes. *Wetlands Ecology and Management* **23**: 327-346.

Phillips S, Rouse GE, Bustin RM. 1997. Vegetation zones and diagnostic pollen profiles of a coastal peat swamp, Bocas del Toro, Panama. *Palaeogeography, Palaeoclimatology, Palaeoecology* **128**: 301-338.
